# Efficacy of Different Cold-Water Immersion Temperatures on Neuromotor Performance in Young Athletes

**DOI:** 10.3390/life12050683

**Published:** 2022-05-05

**Authors:** Jair J. Gaspar-Junior, Rodolfo A. Dellagrana, Fernando S. S. Barbosa, Ana P. Anghinoni, Charles Taciro, Rodrigo L. Carregaro, Paula F. Martinez, Silvio A. Oliveira-Junior

**Affiliations:** 1 Graduate Program in Health and Development in the Midwest Region, Federal University of Mato Grosso do Sul—UFMS, Campo Grande 79070-900, Brazil; gasparjr.ft@gmail.com (J.J.G.-J.); fernando@unir.br (F.S.S.B.); paula.martinez@ufms.br (P.F.M.); 2 Physical Education Department, State University of Ponta Grossa —UEPG, Ponta Grossa 84030-900, Brazil; radellagrana@uepg.br; 3 Graduate Program in Movement Sciences, Federal University of Mato Grosso do Sul—UFMS, Campo Grande 79070-900, Brazil; 4 Department of Education Sciences, Federal University of Rondônia—UNIR, Ariquemes 76872-848, Brazil; 5 School of Physical Therapy, Federal University of Mato Grosso do Sul—UFMS, Campo Grande 79070-900, Brazil; anapaula.anghinoni@gmail.com (A.P.A.); charles.taciro@ufms.br (C.T.); 6 School of Physical Therapy, Master in Rehabilitation Sciences, Universidade de Brasilia—UnB, Brasília 70910-900, Brazil; rodrigocarregaro@unb.br

**Keywords:** muscle strength, fatigue, ice, athletic performance

## Abstract

Cold-Water-Immersion (CWI) has been frequently used to accelerate muscle recovery and to improve performance after fatigue onset. In the present study, the aim was to investigate the effects of different CWI temperatures on neuromuscular activity on quadriceps after acute fatigue protocol. Thirty-six young athletes (16.9 ± 1.4 years-old; 72.1 ± 13.8 kg; 178.4 ± 7.2 cm) were divided into three groups: passive recovery group (PRG); CWI at 5 °C group (5G); and CWI at 10 °C group (10G). All participants performed a fatigue exercise protocol; afterwards, PRG performed a passive recovery (rest), while 5G and 10G were submitted to CWI by means of 5 °C and 10 °C temperatures during 10 min, respectively. Fatigue protocol was performed by knee extension at 40% of isometric peak force from maximal isometric voluntary contraction. Electromyography was used to evaluate neuromuscular performance. The passive recovery and CWI at 5 °C were associated with normalized isometric force and quadriceps activation amplitude from 15 until 120 min after exercise-induced fatigue (F = 7.169, *p* < 0.001). CWI at 5 °C and 10 °C showed higher muscle activation (F = 6.850, *p* < 0.001) and lower median frequency (MF) than passive recovery after 15 and 30 min of fatigue (F = 5.386, *p* < 0.001). For neuromuscular efficiency (NME) recovery, while PRG normalized NME values after 15 min, 5G and 10G exhibited these responses after 60 and 30 min (F = 4.330, *p* < 0.01), respectively. Passive recovery and CWI at 5 °C and 10 °C revealed similar effects in terms of recovery of muscle strength and NME, but ice interventions resulted in higher quadriceps activation recovery.

## 1. Introduction

For decades, the influence of fatigue on human performance has been a widely studied issue in the sport sciences. Fatigue is characterized as an activity-induced decline of muscle performance [1] and may be related to a sequence of events controlling muscle contraction [2], such as reduction in the central nervous system’s drive of active musculature and impaired muscle contractile function [3,4]. Fatiguing events may be compensated for with adequate recovery to reestablish physiological and psychological conditions [5]. Based on this premise, multiple post-exercise recovery interventions have been adopted to restore the musculoskeletal system from fatigue and to compensate for external and internal overloads due to training and competitions [5,6].

Post-exercise cold-water immersion (CWI) has been frequently used to accelerate muscle recovery and to improve performance after fatigue onset [7,8]. Although previous investigations had documented functional performance-adaptive effects in response to different strategies of cooling interventions (e.g., cold packs, cold-water immersion, and ice bags) [9,10,11,12], there is no consensus on the efficacy of these interventions to improve post-exercise recovery. This aspect is relevant because CWI is considered the most popular method in sport practice [13,14,15], and several intervention protocols using temperatures below 15 °C of around 5–15 min duration have been used to improve muscle recovery [16,17]. CWI intervention may provide additional benefits to the recovery immediately after intense activity or muscle damage, which have been associated with post-injury recovery [14,18]. The mechanisms behind the recovery effect derived from cooling methods have been explained by vasoconstrictive effects, followed by a decrease of cell metabolism and inflammation [19]. In addition, CWI presents effects on neural components, as it might inhibit cells regulated by the impulse pain perception through the decrease of acetylcholine concentration, thus reducing neural transmission [20,21].

However, the use of CWI in the context of athletic performance has been challenged, as its effects on muscle electrical activity and performance after fatigue onset remain unclear. Recently, Kodejška et al. [22] demonstrated that CWI generated higher handgrip strength compared with passive recovery in rock-climber athletes. In contrast, Naderi et al. [23] reported that CWI did not lead to a reduction in muscle strength after a single bout of strength training. Contradictory findings related to sequential CWI interventions have also been reported in a recent systematic review [24]. Additionally, the CWI had positive effects in reducing pain, but the effects on restoring muscular function seem to be inconclusive [13]. We raise the question of whether the CWI effects may depend on the water temperature and the time between CWI and the subsequent exercise bout [14,15,25].

Therefore, the aim of this study was to investigate the efficacy of different CWI temperatures (5 °C and 10 °C) on neuromuscular activity of the vastus medialis, rectus femoralis, vastus lateralis, and quadriceps (sum of all muscles) after an acute fatigue protocol. Regarding temperature parameters, 5 °C and 10 °C were the most frequently used in previous studies [10,26,27]. It was hypothesized that CWI with 5 °C would elicit a greater neuromuscular recovery compared with passive recovery (control treatment), due to a more pronounced cold tissue impact [19].

## 2. Materials and Methods

### 2.1. Participants

This is an experimental study and enrolled a sample of 36 healthy 15 to 21 year-old male soccer and basketball practitioners from two different sports teams from Campo Grande, MS, Brazil. Participants were assigned to three age-stratified groups according to sequence admission and post-exercise intervention: (1) passive recovery group (PRG, 17.1 ± 1.7 years-old; 70.2 ± 10.9 kg; 176.9 ± 6.1 cm); (2) CWI at 5 °C group (5G; 17.0 ± 1.5 years; 70.6 ± 9.0 kg; 177.8 ± 8.0 cm); and (3) CWI at 10 °C group (10G; 16.5 ± 1.0 years; 75.7 ± 19.7 kg; 180.5 ± 7.6 cm). The following inclusion criteria were adopted: (1) participants aged 15 to 30 years-old; (2) absence of contraindications to CWI; and (3) be active and performing athletic training and competitions regularly for at least 1 year. Volunteers presenting Raynaud disease, musculoskeletal injuries in lower limbs with retrospective onset within two prior months or being engaged in work activities characterized by high physical effort were excluded. All participants provided written informed consent. Ethical approval was obtained from the local Human Research Ethics Committee (protocol number 2.920.457) and is registered in Registro Brasileiro de Ensaios Clínicos (ReBEC; ID Number: RBR-2z73q5). The impacts of these post-exercise CWI interventions on sensorimotor performance have been published elsewhere [28].

### 2.2. Assessment Procedures

**Figure 1:**
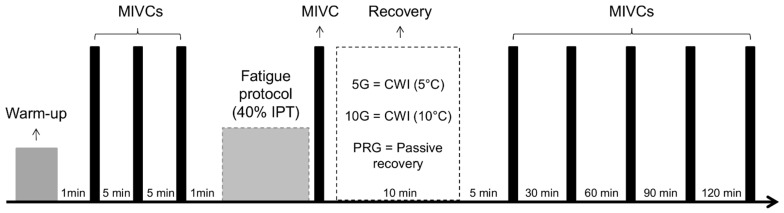
Timeline of the study.

The assessments were performed at two different times, separated by seven days. In the first time interval, anthropometric evaluation, anamneses (injury history, sport practice and physical activity), and a familiarization protocol were performed. During the second interval, each participant performed a brief warm-up based on familiar exercises, and three maximal isometric voluntary contraction (MIVC) tests were made to determine the isometric peak force (IPF). Afterwards, a fatigue protocol was performed by knee extension MIVC at 40% of IPF, followed by cold water immersion (5G and 10G) or passive recovery (PRG). All participants performed the same protocol exercise test; however, PRG performed a passive recovery (rest during 10 min) after testing protocol, while 5G and 10G were submitted to CWI by means of 5 °C and 10 °C temperatures during 10 min, respectively, after the fatigue protocol. After the fatigue protocol, a follow-up period integrated 15-, 30-, 60-, 90- and 120-min intervals of evaluation, in which each participant performed a novel MIVC (Figure 1). On the day before each test, the participants were solicited to avoid vigorous exercises of the lower limbs, alcohol, and/or caffeine intake [28].

### 2.3. Description of the Interventions

The recovery sessions were performed immediately after the MIVC post-fatigue protocol. After performing a MIVC to induce fatigue, participants from PRG remained at rest on a stretcher for 10 min. Regarding the 5G and 10G groups, participants were immersed up to the level of the gonads inside a cold-water plastic receptacle (height, 980 mm; width, 490 mm; length, 560 mm) with total capacity of 240 L. The water cooling was performed using ice cubes, and temperature was controlled by a floating thermometer.

### 2.4. Maximal Isometric Voluntary Contraction (MIVC) Test and Fatigue Induction

Preceding the measurements, a warm-up protocol (knee flexion and extension movements based on familiarization) was performed. Next, each participant sat on a knee extensor device with back adjustments and support, the hip and knee joint positions were maintained at 90° of flexion (0° = full knee extension), and the trunk and knee were firmly strapped to the chair with a seatbelt [29,30,31,32,33]. The test was performed with the dominant limb (preferred limb to kick a ball). After the participant had been appropriately positioned, a load cell of 200 kgf was coupled to the device and was fitted to the subject’s ankle by means of a non-extendable ankle band, in such a way that the force vector was always exerted at 0° in relation to the axis of the load cell. In this position, in which there was restriction of knee movement, three MIVCs were performed for a 6 s period and verbal encouragement was provided to induce the subjects to reach their maximal performance in each trial [34]. A 5 min rest period was taken between trials, and the highest peak force value obtained from the three MIVCs was considered the IPF. Visual feedback of the produced force was provided. After MIVCs, the participants performed a protocol-induced fatigue, and it consisted of an isometric contraction at 40% of IPF. Participants were instructed to maintain the isometric contraction at the target toque level if possible. When the participant could not maintain the target effort level (>5%), the test was interrupted.

### 2.5. Surface Electromyography

Surface electromyography (EMG) analyses were recorded in the MIVCs and submaximal contractions after the fatigue protocol. The EMG signals were recorded using a sampling frequency of 2000 Hz per channel (Miotool, Miotec Equipamentos Biomedicos Ltd.a^®^, Porto Alegre, Brazil). Muscle activation was assessed by means of surface EMG from *vastus lateralis* (VL), *rectus femoris* (RF), and *vastus medialis* (VM). The skin surface was shaved, debrided, and cleaned, followed by attachment of bipolar electrodes (Meditrace, Kendal, Chicopee, MA, USA). The electrodes were placed according to Surface Electromyography for the Non-Invasive Assessment of Muscles (SENIAM) motor point method for positioning the electrodes [35]. Reference electrodes were positioned over the lateral malleolus. The raw EMG data were smoothed using a band-pass filter with frequency range set between 10 and 500 Hz, and notch filter at 60 Hz. The root mean square (RMS) and median frequency (MF) of VL, RF and VM were calculated using the software Miotec Suite, version 1.0. In addition, the RMS sum (VL + RF + VM) and MF average (VL + RF + VM/3) were used to represent muscle activation (time and frequency domain, respectively) of the quadriceps (QUA) [36]. The neuromuscular efficiency (NME) of the VL, RF, VM and QUA muscles was calculated using the ratio of IPF/RMS.

### 2.6. Statistical Analysis

Data normality was verified using a Kolmogorov–Smirnov test. Values are presented as mean and standard deviation (SD). General linear mixed-model analysis (within-subjects factor: time [pre, post, 15, 30, 60, 90 and 120 min] x between factor: group [PRG, 5G and 10G], Two-Way ANOVA) was used to compare the IPF, RMS, MF, and NME. A Bonferroni post hoc test was used for all analysis. All statistical analyses were performed using IBM SPSS Statistics for Windows, version 21.0 (IBM Corp., Armonk, NY, USA). The significance level was set at 5% (*p* < 0.05).

## 3. Results

Table 1 presents the IPF values before and immediately, 15, 30, 60, 90 and 120 min after the fatigue protocol. No group-time interaction (F = 0.496, *p* = 0.915) or group effect (F = 0.476, *p* = 0.626) was found for the current results. However, a significant time effect (F = 7.169, *p* < 0.001) was observed; IPF values were statistically different between pre- and immediately post-fatigue protocol in all groups. Also, 10G presented lower IPF values at 90- and 120-min recovery intervals when compared to the pre-fatigue condition (*p* < 0.05).

**Figure 2:**
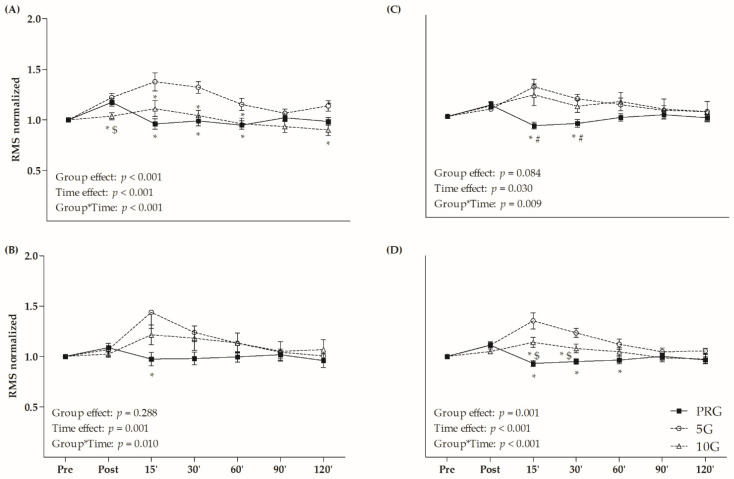
Root mean square (RMS) values normalized by the maximal isometric voluntary contraction (MIVC) in (**A**) *vastus lateralis* (VL); (**B**) *rectus femoris* (RF); (**C**) *vastus medialis* (VM); and (**D**) sum of these muscles (quadriceps–QUA) in different times (post, and 15, 30, 60, 90 and 120 min after fatigue protocol). * *p* < 0.05 versus 5G within time; # *p* < 0.05 versus 10G within time; $ *p* < 0.05 versus PRG within time; Two-Way ANOVA and Bonferroni’s test.

Figure 2 presents the RMS values normalized by IPF at the pre-fatigue time. Significant group × time interactions were observed for VL (F = 4.631, *p* < 0.001), RF (F = 2.936, *p* = 0.010), VM (F = 3.077, *p* = 0.009) and QUA (F = 6.850, *p* < 0.001) analyzes. In general, the 5G presented higher RMS values than PRG and 10G in the 15-, 30- and 60-min times for VL and QUA. Relative to the RF, the 5G showed higher RMS values than PRG at 15 min. Considering VM analysis, the PRG presented lower RMS values than 5G and 10G at the 15- and 30-min times. In general, 5G presented higher RMS for QUA than other groups at 15 and 30 min after protocol, as well as higher RMS than PRG at 60 min (*p* < 0.05). Regarding the time, 5G and PRG exhibited increased RMS of quadriceps immediately after protocol (Figure 2D), but only 5G maintained the higher RMS at 15- and 30-min after fatigue (*p* < 0.05). No time effect was observed for 10G.

**Figure 3:**
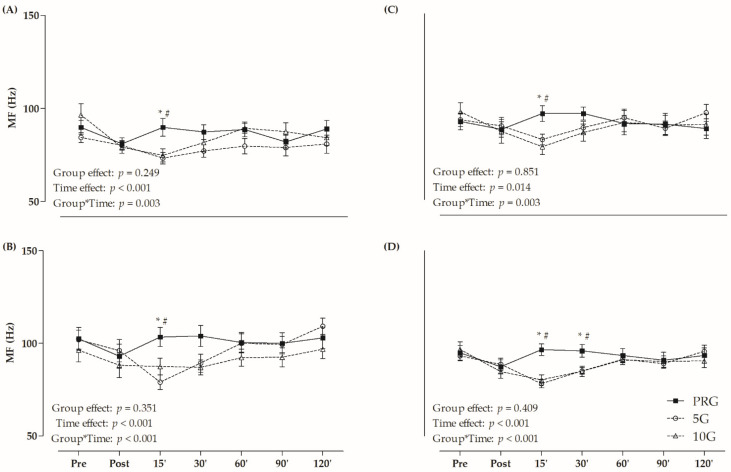
Median of frequency (MF) values in (**A**) *vastus lateralis* (VL); (**B**) *rectus femoris* (RF); (**C**) *vastus medialis* (VM); and (**D**) average of these muscles (quadriceps–QUA) in different times (post, and 15, 30, 60, 90 and 120 min after fatigue protocol). * *p* < 0.05 versus 5G within time; # *p* < 0.05 versus 10G within time; Two-Way ANOVA and Bonferroni’s test.

The values of MF are presented in Figure 3. A significant group and time interaction effect was observed for VL (F = 2.622, *p* = 0.003), RF (F = 3.482, *p* < 0.001), VM (F = 2.619, *p* = 0.003), and QUA (F = 5.386, *p* < 0.001) analyses. In relation to VL, RF, and VM, PRG presented higher MF than 5G and 10G at 15-min. PRG presented higher MF values than 5G and 10G at 15 and 30 min after fatigue protocol for QUA. Furthermore, only 5G showed lower MF after 15 min in comparison to the initial value for RF and QUA (*p* < 0.05), while 10G had lower MF immediately, 15 and 30 min after exercise compared to the baseline time for VL and QUA (*p* < 0.05).

Neuromuscular efficiency (NME) results are exhibited in Table 2. There was a statistically significant interaction between group and time for VL (F = 10.814, *p* < 0.01), RF (F = 2.224, *p* = 0.031), VM (F = 2.924, *p* = 0.005), and QUA (F = 4.330, *p* < 0.01). In the VL analysis within PRG, NME was higher at the pre (start), 15-, and 60-min interval in comparison to the post (exhaustion) interval. Considering 5G, pre and 90-min intervals were associated with increased NME values compared to other intervals. Regarding RF, 5G presented higher initial NME in relation to the measures from 15 and 30 min of evaluation; also, NME measures were lower at the 15- than the 90- and 120-min intervals. Within 10G, NME was higher at the initial time (pre) in comparison to post, 15-, 30- and 60-min intervals. In relation to VM, PRG exhibited higher NME during 15 and 30 min when compared to the post interval (exhaustion). 5G and 10G presented higher initial NME in comparison to the post, while 5G exhibited greater initial NME when compared to the 15- and 30-min intervals. For QUA, the PRG presented higher NME in the pre than post, while at 15, 30 and 60 min, higher NME was observed compared to the post-fatigue protocol. 5G showed higher NME pre than post, 15 and 30 min, as well higher NME at 90 and 120 min compared to 15 min. Finally, 10G presented higher NME at the pre than post and 15-min intervals.

## 4. Discussion

The aim of the current study was to verify the effects of different CWI temperatures on isometric peak force and muscular activity of the quadriceps muscles (VL, RF, VM and QUA) after exercise-induced fatigue. We hypothesized that a lower temperature (5 °C) protocol would be more effective for muscular recovery (i.e., force and muscle activation). According to the results, passive recovery and both CWI protocols (5 °C and 10 °C) can attenuate the fatigue (IPF and RMS values) and revealed similar effects. Based on the RMS values, 5 °C intervention was able to increase the quadriceps muscle activity after exercise- induced fatigue (post, 15- and 30-min). On the other hand, regarding MF values, both CWI protocols promoted a brief reduction in the frequency of quadriceps muscle activity and had similar effects at the end of the 120-min period. Furthermore, 5G and 10G presented a delay in the NME recovery, since NME values after 15- and 30-min of fatigue were lower than pre-protocol (baseline) for 5G and 10G, respectively.

In general, athletes make excessive efforts without sufficient time for adequate muscle recovery; thus, the effects of cooling interventions (especially CWI) on recovery have been widely investigated in the literature [14,37,38,39]. Likewise, muscle fatigue has been assessed through isometric force fall in response to MIVCs performed before and after different exercises protocols [1,40,41]. In the present study, the isometric exercise bout at 40% of IPF was able to induce a fatigue condition, as evidenced by the reduced isometric strength immediately after exercise (Table 1). While PRG and 5G presented similar effects in terms of isometric strength recovery, 10G showed reduced levels of muscle strength after 120 min from fatigue onset. Comparatively, these findings partially controverted the conclusions of other studies, which reported effects in isometric force recovery in response to CWI [37,38]. Vieira et al. [37] showed that CWI (20 min at 5 °C or 15 °C) was not effective in accelerating the isometric strength recovery in the long term (i.e., >24 h) for knee extensors. Also, according to Argus et al. [38], CWI intervention (14 min at 15 °C) did not improve the short term (i.e., <4 h) isometric force recovery of knee extensors, corroborating our results about 10 °C intervention.

Probably, these discrepancies might be associated with different procedures or timing of CWI application and different methods used to assess muscle effects. Some aspects might explain these divergences, such as the exercise protocol used to induce the fatigue, since both aforementioned studies [37,38] used dynamic contractions (i.e., resistance training session and countermovement jump) as fatigue protocol, while an isometric exercise protocol (40% of IPF) was applied in the present study. A long-term cooling intervention applied after exercise-induced damage may promote beneficial effects on muscle damage [42,43]. However, acute cooling methods have been associated with inconclusive results [39]. Deleterious effects of cooling have been observed for neuromuscular parameters (e.g., nerve conduction velocity and firing rate) [44,45], which could be associated with impaired sensorimotor performance [28]. Therefore, the results of acute interventions in muscle activity recovery remain unclear. 

Regarding acute cooling intervention applied before exercise, studies have reported decreased muscle activation (EMG amplitude) in response to the cold intervention [45,46]. Other investigators reported no significant changes [47] or increased muscle activation [48]. Differences relative to muscle contractions, cooling methods, cooling temperatures, and anatomical sites of cooling intervention seem to explain contradictory results among several studies. Diverse cooling methods (e.g., ice pack, CWI and ice massage) are effective in reducing skin temperature, but potential effects on neuromuscular system differ among methods [49]. Besides, cooling temperatures presented a wide variety in the literature, in which temperatures between 5 °C and 20 °C have been used by authors [20,37]. As a result, studies that aimed to verify effects of cooling methods on muscle recovery have shown conflicting results.

Reductions in RMS values after exercise-induced fatigue have been reported in the literature [38,39]. This phenomenon is possibly associated with accumulation of metabolites (e.g., ADP, Ca^+^, H^+^ and lactate), followed by inhibition of motor neurons [50] or reduction of supraspinal descending drive [51]. Inversely, the results showed that passive recovery and CWI with temperatures at 5 °C and 10 °C were able to attenuate the neuromuscular fatigue; however, only CWI at 5 °C increased quadriceps activation after the fatigue protocol (Figure 2). The increases of EMG amplitude (RMS) could be related to more muscle fiber recruitment to perform a given workload. Therefore, it is possible that the nervous system recruited more muscle fibers, especially faster fibers, in order to compensate for the suppressed muscle function caused by low temperatures [52].

The MF is a neuromuscular variable often used to estimate peripheral fatigue during isometric contractions [53]. In the current study, CWI with temperatures of 5 °C and 10 °C provoked reductions in MF within 15 min after protocol, indicating reduced neuromuscular firing rate (Figure 3). Similarly, Kim and Hurr [39] verified decreasing MF in the first 10 sec. of the Wingate test with cooling suit recovery (7 °C). Other studies have already shown decreased MF values after cooling intervention [45,48]. According to Herrera et al. [49], reduced muscular temperature derived from cooling methods may be associated with slower nerve conduction velocity, which could increase the duration of action, potentially affecting the MF values. They claimed that CWI is the main cooling method to inhibit the neuromuscular transmission (i.e., nerve conduction velocity), reducing the capacity to perform tasks related to maximal force, but presenting therapeutic effects, such as control of pain and muscle spasms [49].

Importantly, the NME is the relationship between neural stimulus and capacity to generate force. Therefore, improvements in the NME are associated with support of high loads by muscle and lower neural recruitment [54]. In the present study, 5G and 10G showed a delay in the NME recovery of quadriceps (Table 2), sustaining increased neural recruitment to achieve an equal amount of muscle strength 15 and 30 min after fatigue onset. Indeed, 5G and 10G exhibited higher RMS values than PRG (Figure 2), which may have impacted NME recovery after CWI. In response to the cold temperatures (i.e., approximately <15 °C), muscle force generating ability may decline, and as muscles attempt to maintain the force capacity, they recruit higher threshold motor units earlier [52].

A limitation that could be pointed out in our study concerns the inflammatory markers and perceived responses to complement the results of muscle recovery. However, the aim of the present study was to verify neuromuscular recovery, which is attributed to the EMG analysis performed here. Also, the skin temperature was not assessed here, but a difference of up to 10 °C between skin and muscle temperatures has been observed in the literature; thus, extrapolating the muscle cooling to the skin temperature may be not adequate [55]. Our results are limited to young athletes and for exercises with isometric contractions. Future research could verify the effect of different cooling methods and temperatures on neuromuscular recovery after isotonic or isometric exercises to induce fatigue. The understanding of optimal cooling parameters (e.g., method, temperature, and time of application) is essential to provide guidelines for muscle recovery.

Despite this, the current findings are of interest to sports science practitioners and medical teams who have considered using passive recovery or CWI interventions to improve physiological restoration of young athletes. Regarding the temperature of cooling methods to recovery young athletes, here, CWI at 5 °C and 10 °C increased quadriceps activation during the initial 15 to 60-min interval after fatigue onset. From an athletic recovery perspective, these findings expand our understanding of the effectiveness of CWI interventions in healthy and trained young athletes to improve recovery during breaks and interval periods in soccer or basketball matches.

## 5. Conclusions

Our findings showed that 5 °C and 10 °C CWI interventions were not more effective than passive recovery on isometric muscle strength and neuromuscular efficiency recovery after 120 min of muscle fatigue onset. On the other hand, both CWI interventions were more effective in quadriceps activation recovery than passive recovery intervention.

## Figures and Tables

**Table 1:** Mean and standard deviation of isometric peak force (kgf) from isometric voluntary contraction (MIVC) according to group (Gr) and time (Ti) of evaluation (15, 30, 60, 90 and 120 min) after fatigue protocol.

Group	Time of Evaluation							*p*-Value		
	Pre	Post	15-min	30-min	60-min	90-min	120-min	Gr	Ti	Gr × Ti
PRG	43.5 ± 8.9	39.8 ± 9.4 *	41.2 ± 10.3	41.9 ± 11.3	42.9 ± 12.6	41.5 ± 10.9	39.8 ± 11.3			
5G	47.4 ± 6.4	42.1 ± 4.8 *	44.7 ± 6.2	45.8 ± 5.4	44.8 ± 6.4	44.8 ± 4.4	44.2 ± 4.5	0.626	<0.001	0.915
10G	46.5 ± 10.1	40.6 ± 7.7 *	44.2 ± 11.4	42.9 ± 9.7	43.8 ± 9.8	41.9 ± 8.9 *	41.8 ± 10.3 *			

PRG, passive recovery group; 5G, cold water immersion at 5 °C group; 10G, cold water immersion at 5 °C group. * *p* < 0.05 versus Pre within group; Two-Way ANOVA and Bonferroni’s test.

**Table 2:** Mean and standard deviation of neuromuscular efficiency for *vastus lateralis* (VL), *rectus femoris* (RF), *vastus medialis* (VM) and sum of these muscles (quadriceps, QUA) according to group (Gr) and time of evaluation (15, 30, 60, 90 and 120 min) after fatigue protocol.

Muscle	Gr	Time						
		Pre	Post	15-min	30-min	60-min	90-min	120-min
	PRG	0.127 ± 0.043	0.099 ± 0.035 *	0.127 ± 0.049 ^#^	0.127 ± 0.056 ^#^	0.139 ± 0.080 ^#^	0.120 ± 0.050	0.120 ± 0.051
VL	5G	0.153 ± 0.043	0.112 ± 0.030 *	0.107 ± 0.031 *	0.112 ± 0.027 *	0.127 ± 0.035 *	0.137 ± 0.037 ^#$‡^	0.128 ± 0.038 *
	10G	0.149 ± 0.065	0.126 ± 0.059	0.129 ± 0.065	0.133 ± 0.061	0.146 ± 0.066	0.142 ± 0.055	0.150 ± 0.070
	PRG	0.118 ± 0.052	0.100 ± 0.046	0.116 ± 0.049	0.119 ± 0.058	0.120 ± 0.066	0.109 ± 0.039	0.116 ± 0.058
RF	5G	0.139 ± 0.044	0.116 ± 0.034	0.095 ± 0.029 *	0.110 ± 0.033 *	0.117 ± 0.032	0.127 ± 0.036 ^$^	0.130 ± 0.041 ^$^
	10G	0.157 ± 0.114	0.130 ± 0.084 *	0.119 ± 0.080 *	0.123 ± 0.083 *	0.129 ± 0.078 *	0.134 ± 0.084	0.132 ± 0.093
	PRG	0.132 ± 0.086	0.109 ± 0.069	0.138 ± 0.088 ^#^	0.140 ± 0.099 ^#^	0.128 ± 0.071	0.122 ± 0.073	0.121 ± 0.077
VM	5G	0.162 ± 0.050	0.136 ± 0.040 *	0.121 ± 0.036 *	0.134 ± 0.034 *	0.140 ± 0.042	0.147 ± 0.042 ^$^	0.145 ± 0.042
	10G	0.149 ± 0.088	0.115 ± 0.051 *	0.124 ± 0.067	0.124 ± 0.056	0.126 ± 0.063	0.134 ± 0.077	0.139 ± 0.092
	PRG	0.039 ± 0.012	0.032 ± 0.012 *	0.040 ± 0.015 ^#^	0.041 ± 0.019 ^#^	0.041 ± 0.019 ^#^	0.038 ± 0.015	0.037 ± 0.014
QUA	5G	0.049 ± 0.012	0.039 ± 0.010 *	0.034 ± 0.008 *	0.038 ± 0.008 *	0.041 ± 0.009	0.044 ± 0.010 ^$‡^	0.043 ± 0.012 ^$^
	10G	0.047 ± 0.025	0.039 ± 0.019 *	0.040 ± 0.022 *	0.041 ± 0.020	0.042 ± 0.021	0.043 ± 0.022	0.045 ± 0.026

PRG, passive recovery group; 5G, cold water immersion at 5 °C group; 10G, cold water immersion at 5 °C group. Effects within group: * *p* < 0.05 vs. pre; ^#^ *p* < 0.05 vs. post; ^$^ *p* < 0.05 vs. 15-min; ^‡^ *p* < 0.05 vs. 30-min within group; Two-Way ANOVA and Bonferroni’s test.

## Data Availability

The code and models will be available upon request to authors and under restrictions regarding ethical aspects.
